# A copper-iodide cluster microcube-based X-ray scintillator

**DOI:** 10.1038/s41377-023-01273-5

**Published:** 2023-09-20

**Authors:** Jian Qiu, Xiaogang Liu

**Affiliations:** 1 Joint School of the National University of Singapore and Tianjin University, International Campus of Tianjin University, Binhai New City, 350207 Fuzhou, China; 2 Department of Chemistry, National University of Singapore, Singapore, 117543 Singapore; 3 Institute of Materials Research and Engineering, Agency for Science, Technology and Research, Singapore, 138634 Singapore

**Keywords:** Imaging and sensing, Nanoparticles

## Abstract

Newly developed copper-iodide cluster microcubes offer a solution to the issues commonly faced by powder scintillation screens. These problems include inadequate scintillation performance and significant light scattering, resulting in poor image quality. With the advent of monodisperse copper-iodide cluster microparticle scintillators, efficient and long-term stable scintillation is achieved, while ensuring biocompatibility. Moreover, they enable high-resolution static and dynamic X-ray imaging, providing high image quality.

X-ray imaging has revolutionized medical diagnostics, nondestructive testing, security screening, and materials research^1^. Discovered over a century ago, X-rays are a form of high-energy electromagnetic radiation that can penetrate various materials. The ability to capture internal structures and details invisible to the naked eye makes X-ray imaging an invaluable tool for diverse applications. However, the poor quality of X-ray images, characterized by low contrast and weak spatial resolution, has hindered the progress of X-ray imaging technologies. In particular, achieving clear, stable, and high-resolution green X-ray imaging remains a significant challenge.

Scintillators, which are essential components of X-ray imaging systems, can convert ionizing radiation into visible photons to improve image contrast and resolution. High imaging contrast typically relies on scintillators with high sensitivity and strong radioluminescence. Superior spatial resolution can be achieved with small, uniform scintillators with minimal light scattering. Moreover, scintillators must be robust, environmentally friendly, and biocompatible to ensure long-term usability and safety in biomedical X-ray imaging. The demand for high-quality X-ray imaging drives the search for scintillators with enhanced sensitivity, efficient radioluminescence, stability, low toxicity, and uniform particle size distribution. For example, inorganic perovskite nanocrystal scintillators exhibit strong X-ray stopping power and efficient radioluminescence, but their toxic, unstable, and self-absorbing nature poses challenges^2,3^. Organic scintillators offer flexibility and biocompatibility but lack sensitivity to X-rays^4^.

Now, writing in *Light Science & Application*, Wang et al.^5^ from Northwestern Polytechnical University report the synthesis of monodisperse copper-iodide cluster microcubes with precise kinetic control of crystallization. These microcubes serve as ‘quasi-ideal’ scintillators with high sensitivity, bright radioluminescence, long-term stability, eco-friendliness, and homogeneous size distribution. As a result, they enable high-quality static and real-time X-ray imaging, as shown in Fig. 1.

**Fig. 1:**
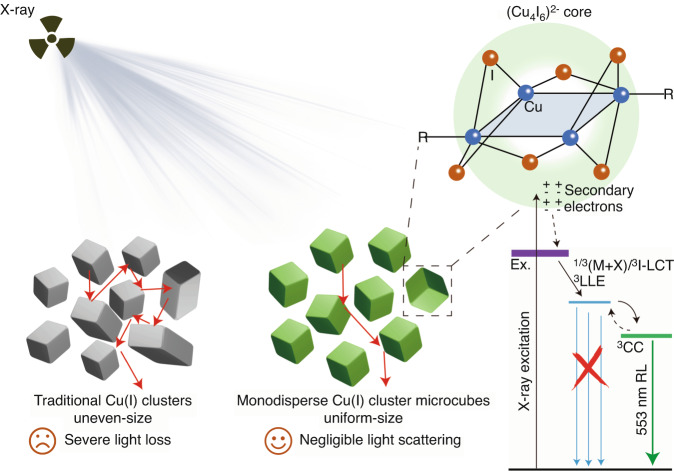
Schematic representation of X-ray-excited monodisperse copper-iodide cluster microcubes with uniform size distribution. These microcubes emit 535 nm radioluminescence originating from triplet cluster-centered excited state (^3^CC) and show negligible light scattering

The principle of X-ray imaging is based on the differential absorption of X-rays by objects with varying density and thickness. This leads to different amounts of X-rays reaching the scintillation screen, forming an image pattern through unequal radioluminescence intensity by scintillators. Scintillation involves three stages: conversion, transport, and emission^6^.

During the conversion stage, emitters absorb X-rays, generating numerous secondary electrons through electron scattering and Auger processes. These high-energy secondary electrons then interact with phonons, producing low-kinetic-energy electrons and holes. Efficient conversion heavily relies on the absorption of X-rays by the emitters, which is proportional to the fourth power of their effective atomic number (*Z*_eff_). To ensure efficient X-ray absorption, the heavy atom effect becomes essential.

In their work, Wang et al.^5^ design a tetrakis-copper-iodide cluster called Cu_4_I_6_(pr-ted)_2_ (where pr-ted represents 1-propyl-1,4-diazabicyclo[2.2.2]octan-1-ium). Leveraging the heavy atom effect of Cu and I, the designed Cu_4_I_6_(pr-ted)_2_ cluster exhibited a *Z*_eff_ of 46.5. This value surpasses that of the commercial bulk scintillator YAlO_3_:Ce and is comparable to halide perovskite scintillators, enabling it to efficiently absorb X-rays and exhibit high sensitivity in response to X-ray irradiation.

After conversion, generated electrons and holes move to the emitting center, but defects in scintillators can trap them, leading to nonradiative energy loss. To enhance radioluminescence efficiency, optimized crystallinity is crucial to suppress the formation of bulk traps and surface defects. Cu(I) clusters exhibit good intrinsic stability due to their coordination bonds^7^, but external factors such as moisture, heat, and radiation can introduce traps and defects. Notably, Cu(I) clusters are known to be easily oxidized to Cu^0^ and I^0^ vacancies in the atmosphere, or form Cu and I interstitials^8^.

To investigate these issues in Cu_4_I_6_(pr-ted)_2_, Wang et al.^5^ conduct density functional theory calculations to study the formation energy for different defects. They find that Cu interstitials have the lowest formation energy, prompting them to anneal Cu_4_I_6_(pr-ted)_2_ at 200 °C in N_2_ to improve crystallinity. The annealed Cu_4_I_6_(pr-ted)_2_ manifests enhanced stability, outperforming Pb and Cu-based halide perovskite scintillators.

Improved crystallinity also enhances radioluminescence emission from the triplet cluster-centered (^3^CC) excited state, comparable to commercial scintillators and Cu-based perovskite scintillators. Cu_4_I_6_(pr-ted)_2_ scintillators exhibit a low X-ray detection limit of 22 nGy_air_ s^−1^, making them promising for X-ray imaging.

The merits of Cu_4_I_6_(pr-ted)_2_ as an X-ray scintillator can be generalized to most Cu(I) clusters due to their strong X-ray absorption, efficient radioluminescence, long-term stability, and eco-friendliness. However, not all Cu(I) clusters achieve the same high-quality X-ray imaging as demonstrated by Wang and co-workers^5^. This is because conventional synthesis methods lack precise control over crystallization kinetics, resulting in irregular morphologies, non-uniform size distribution, poor dispersity of powders, and strong light scattering in scintillation films^9,10^.

In contrast, Wang et al.^5^ employ hot injection to precisely control crystallization kinetics, leading to monodisperse copper-iodide cluster microcubes with an average size of 2.2 µm. These microcubes exhibit negligible light scattering, enabling exquisite static X-ray imaging with spatial resolution up to 20 lp mm^−1^. Moreover, the authors showcase real-time dynamic X-ray imaging with these copper-iodide cluster microcubes, showing no ghosting effect at an imaging rate of 6.45 frames per second.

The synthesis and application of copper-iodide cluster microcube scintillators through hot injection present exciting avenues for high-quality X-ray imaging. However, further investigation is needed to fully explore their potential. One area of inquiry is the universality of hot injection as a synthesis method for other monodisperse Cu(I) cluster systems. In the context of dynamic X-ray imaging, a major drawback of Cu(I) cluster scintillators is their emission mainly coming from triplet ^3^CC states with long lifetimes, ranging from microseconds to milliseconds. To expand ultra-fast X-ray imaging, this issue needs to be addressed in future research. One possible strategy to address this challenge is to couple Cu(I) cluster scintillators with plasmonic arrays^11^, which may offer innovative solutions to enhance their performance. Nevertheless, the study of copper-iodide clusters as scintillators and their application in real-time X-ray imaging holds great promise. This cutting-edge research inspires a new direction in the field and opens the door for the development of other luminescent clusters such as silver clusters^12^, gold clusters^13^, and even complexes of rare-earth ions^14^, into X-ray scintillation and detection systems.

## References

[1] Ou XY (2021). Recent development in X-ray Imaging technology: future and challenges. Research.

[2] Chen QS (2018). All-inorganic perovskite nanocrystal scintillators. Nature.

[3] Yi LY (2023). X-ray-to-visible light-field detection through pixelated colour conversion. Nature.

[4] Gan N (2022). Organic phosphorescent scintillation from copolymers by X-ray irradiation. Nat. Commun..

[5] Wang YZ (2023). Efficient X-ray luminescence imaging with ultrastable and eco-friendly copper(I)-iodide cluster microcubes. Light Sci. Appl..

[6] Wu Y (2023). Halide perovskite: a Promising candidate for next-generation X-ray detectors. Adv. Sci..

[7] Troyano J, Zamora F, Delgado S (2021). Copper(I)-iodide cluster structures as functional and processable platform materials. Chem. Soc. Rev..

[8] Hu QS (2023). Highly effective hybrid copper(I) iodide cluster emitter with negative thermal quenched phosphorescence for X-ray imaging. Angew. Chem. Int. Ed..

[9] Zhang N (2023). Intramolecular charge transfer enables highly-efficient X-ray luminescence in cluster scintillators. Nat. Commun..

[10] Zhao WJ (2023). Color-tunable and stable copper iodide cluster scintillators for efficient X-ray imaging. Adv. Sci..

[11] Wu YM (2019). Upconversion superburst with sub-2 μs lifetime. Nat. Nanotechnol..

[12] Yang JS (2020). Extra silver atom triggers room-temperature photoluminescence in atomically precise radarlike silver clusters. Angew. Chem. Int. Ed..

[13] Huang RW (2023). Radioluminescent Cu-Au metal nanoclusters: synthesis and self-assembly for efficient X-ray scintillation and imaging. J. Am. Chem. Soc..

[14] Liu XM (2023). Lanthanide(III)-Cu_4_I_4_ organic framework scintillators sensitized by cluster-based antenna for high-resolution X-ray imaging. Adv. Mater..

